# My Last Amalgam Filling

**Published:** 1851-04

**Authors:** 


For the Dental Register of the West.

### MY LAST AMALGAM FILLING.

The dentist, after thoroughly qualifying himself for the practice of his profession, for which the dental colleges of our country furnish ample facilities, should avail himself of all the means within his reach to add to his stock of knowledge relative to the treatment of the diseases of the dental organs and the parts intimately connected with them, including those of rare as well as common occurrence. For this purpose he should procure all valuable works on the subject when they are published and be a subscriber to one or more of the four excellent dental journals sustained by the profession in the United States. He could not fail to be remunerated one hundred fold for the trivial expense thus incurred. A sense of duty to his patrons, as well as a most commendable desire to excel in his profession, should prevent him from considering any amount of money expended to acquire such information as ill spent.

The dentist may gain much valuable information by watching the results of his own operations. Although he may not experiment rashly on his patient, he may perform operations in such various modes as are sanctioned by experience and a sound judgment. For example, he may prepare his gold for filling teeth by folding and cutting it in strips, or by folding and twisting it, or by twisting and cutting it into blocks; and while he may make an excellent filling with his gold prepared by either of these modes, he will, no doubt, after experimenting so as to enable him to form an intelligent opinion as to the merits of each, prefer one of them to the others; or, perhaps, he may learn that he can make the best filling with the blocks in most cases, but that for cavities of a peculiar kind the strips are more desirable. Or in plate work, he may split the rivets of the teeth, or take them out of the plaster and press them down with the punch or hammer previous to soldering; and a few trials will satisfy him respecting the mode most convenient for him to adopt.

The dentist may sometimes find it advantageous to examine the operations of others, profiting by their improvements and avoiding their obvious defects. He will find comparing notes with his professional brethren a valuable means of improvement. For this the meetings of our dental societies afford excellent opportunities.

The dentist should not only be thoroughly acquainted with the best modes of performing the varied and complicated operations entrusted to his care, but he should be able to decide as the *material* proper to be used in different cases. Does palladium answer so well for sustaining artificial teeth as to render it a desideratum for those who are illy able to bear the expense of gold for this purpose? Is it admissable to fill large cavities in the posterior teeth for this class of patients with tin instead of gold? Is it proper to use amalgam for teeth so badly decayed as to render it impossible to fill them with foil? Or is it right to fill teeth with amalgam when it is probable that the use of foil would prove less successful in preserving them? These and many other questions that might be asked, the dentist should be ready to answer intelligently.

Respecting amalgam, we of the Mississippi Valley Association of Dental Surgeons unanimously resolved, some half a dozen years ago, that we would not use it ourselves, nor countenanace its use by others. This resolution was passed probably from a desire on the part of every member present to separate ourselves so far as possible, in faith and practice, from a certain class of operators who made a free and most reprehensible use of amalgam.

About eight years ago the writer of this filled twelve or fifteen badly decayed teeth, with their vitality destroyed, with amalgam; always telling the patients that it was not a good article for filling teeth, and that it would answer only a temporary purpose for such teeth as were not worth filling with gold. No bad results followed, but their blackened surfaces, and the discolored teeth, with a dislike to employ a substance in so bad repute, and used so extensively by empirics, led to its abandonment. The last case was that of a superior molar with the anterior portion including the grinding surface below it gone, presenting a cavity of no very desirable form. Unwilling to use amalgam the tooth was filled with gold, rather to satisfy the patient who had been told by another dentist that it could be done, than from the expectation of its doing any good. So much of the anterior part of the tooth was wanting that it was impossible to give the cavity a shape to retain the foil, and it soon began to come out; when, having endeavored in vain to persuade the patient to have it removed, at his urgent request, as a dernier resort, it was filled with amalgam, with the expectation that it would prove beneficial but a few months. In this the operator was agreeably disappointed, for when he last saw the patient a few months ago, the filling was doing well, and had given the patient no trouble during the seven years it had been worn. If this tooth could have been filled with gold so as to have had it retained, it might not have resisted the action of the food in mastication so well, on account of the extent of surface exposed to it. Was the employment of amalgam in this case morally wrong? Would it be wrong to use it in similar cases, for teeth whose fangs were sufficiently healthy to warrant it, but their crowns too badly decayed to admit of their being filled with foil; or for teeth decayed along the edges of the gums caused by wearing clasps, since in the opinion of some operators of experience it preserves such teeth better than any material that can be employed?

Enquirer.

